# The investigation of oncolytic viruses in the field of cancer therapy

**DOI:** 10.3389/fonc.2024.1423143

**Published:** 2024-07-10

**Authors:** Zijun Yuan, Yinping Zhang, Xiang Wang, Xingyue Wang, Siqi Ren, Xinyu He, Jiahong Su, Anfu Zheng, Sipeng Guo, Yu Chen, Shuai Deng, Xu Wu, Mingxing Li, Fukuan Du, Yueshui Zhao, Jing Shen, Zechen Wang, Zhangang Xiao

**Affiliations:** ^1^ Gulin Traditional Chinese Medicine Hospital, Luzhou, China; ^2^ Laboratory of Molecular Pharmacology, Department of Pharmacology, School of Pharmacy, Southwest Medical University, Luzhou, China; ^3^ Research And Experiment Center, Sichuan College of Traditional Chinese Medicine, Mianyang, China; ^4^ Cell Therapy & Cell Drugs of Luzhou Key Laboratory, Luzhou, China; ^5^ South Sichuan Institute of Translational Medicine, Luzhou, China; ^6^ Department of Pharmacology, School of Pharmacy, Sichuan College of Traditional Chinese Medicine, Mianyang, China

**Keywords:** oncolytic viruses, cancer therapy, genetic engineering, combination therapy, clinical trials

## Abstract

Oncolytic viruses (OVs) have emerged as a potential strategy for tumor treatment due to their ability to selectively replicate in tumor cells, induce apoptosis, and stimulate immune responses. However, the therapeutic efficacy of single OVs is limited by the complexity and immunosuppressive nature of the tumor microenvironment (TME). To overcome these challenges, engineering OVs has become an important research direction. This review focuses on engineering methods and multi-modal combination therapies for OVs aimed at addressing delivery barriers, viral phagocytosis, and antiviral immunity in tumor therapy. The engineering approaches discussed include enhancing *in vivo* immune response, improving replication efficiency within the tumor cells, enhancing safety profiles, and improving targeting capabilities. In addition, this review describes the potential mechanisms of OVs combined with radiotherapy, chemotherapy, cell therapy and immune checkpoint inhibitors (ICIs), and summarizes the data of ongoing clinical trials. By continuously optimizing engineering strategies and combination therapy programs, we can achieve improved treatment outcomes and quality of life for cancer patients.

## Graphical Abstract

OV, oncolytic virus; scFV, single-stranded fragment variable; ICIs, immune checkpoint inhibitors; MSC, mesenchymal stem cell; TILs, tumor-infiltrating lymphocytes.

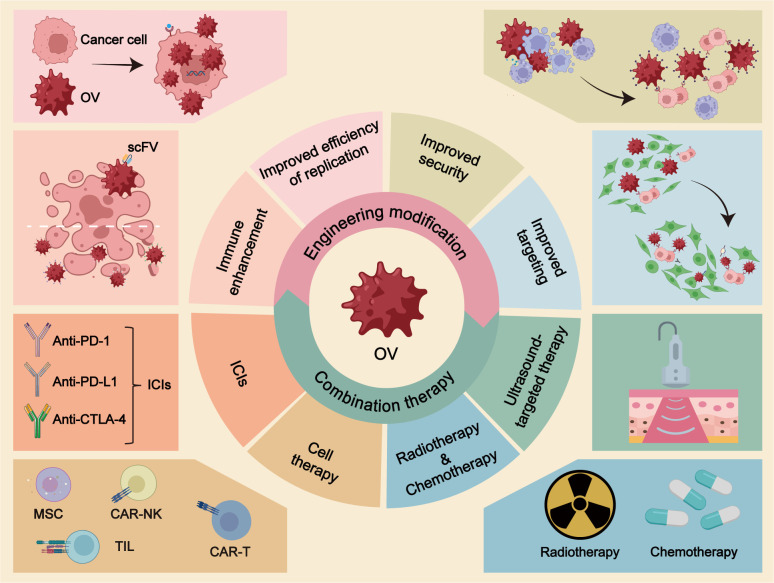

## 1 Introduction

Oncolytic viruses (OVs) are a specific type of viruses that can selectively replicate within tumor cells, inducing apoptosis while also stimulating the immune response and preserving normal tissue from destruction (1). Over the past two decades, extensive research in genetic engineering, tumor immunology, and molecular biology has established oncolytic virus (OV) therapy as a promising approach for cancer treatment (2, 3). OVs can be categorized into two main groups: naturally occurring viruses and genetically modified viruses (4). Naturally occurring OVs include reovirus, newcastle disease virus (NDV), myxoma virus (MYXV; Poxvirus), and seneca valley virus (SVV), while most OVs have been genetically modified or serve as vectors, including measles virus (MV; Paramyxovirus), poliovirus (PV; Picornavirus), vaccinia virus (VV; Poxvirus), adenovirus (Ad), and herpes simplex virus (HSV). Genetic modifications aim to enhance the targeting specificity and safety of the OV towards tumor cells by improving selective replication and cleavage capabilities, and augmenting host anti-tumor immunity levels (1, 5, 6).

With the application of spatial transcriptomics (7), single-cell RNA sequencing (scRNA-seq), and proteomics technology in cancer research (8–10), the significance of tumor microenvironment (TME) in cancer biology has been recognized. Cancer is a complex evolutionary and ecological process involving interactions between tumor cells and TME (11). The complexity and heterogeneity of TME are closely associated with tumor growth, metastasis, and response to therapy, making it a crucial target for cancer treatment (12). Although OVs have emerged as a potential therapeutic option for cancer due to their precise targeting ability, high killing rate, dose escalation over time, and minimal side effects; however, using a single type of OV alone is insufficient to overcome the challenges posed by the immunosuppressive TME resulting in limited anti-tumor effects (13). Therefore, this review aims to summarize engineering modifications of OVs and multi-modal combination therapies that can address delivery barriers, viral phagocytosis issues, antiviral immunity responses along with other challenges faced by OV-based cancer therapy (14–16). Additionally, we will introduce clinical data from current major studies on OV.

## 2 Engineering modification of OVs

### 2.1 Enhancement of OVs immune response *in vivo*

The immune response of OVs is a crucial mechanism in tumor treatment. Enhancing the *in vivo* immune response of OVs is a complex process involving multiple aspects of optimization and strategic approaches. It has been reported that the regulation of the following aspects can break through the immune system barrier and improve the *in vivo* immune response to OVs (1): enhancing T cell activation, polarization, and memory T cell generation (2); inhibiting cancer immune escape through cytokines and blocking immunosuppressive ligands (3); disrupting physical barriers and increasing immune cell infiltration (4); suppressing immunosuppressive cells (17). In this process, immune checkpoint molecules and various cytokines in the TME play pivotal roles.

#### 2.1.1 Engineered OVs carrying immune checkpoint molecules

**Figure 1:**
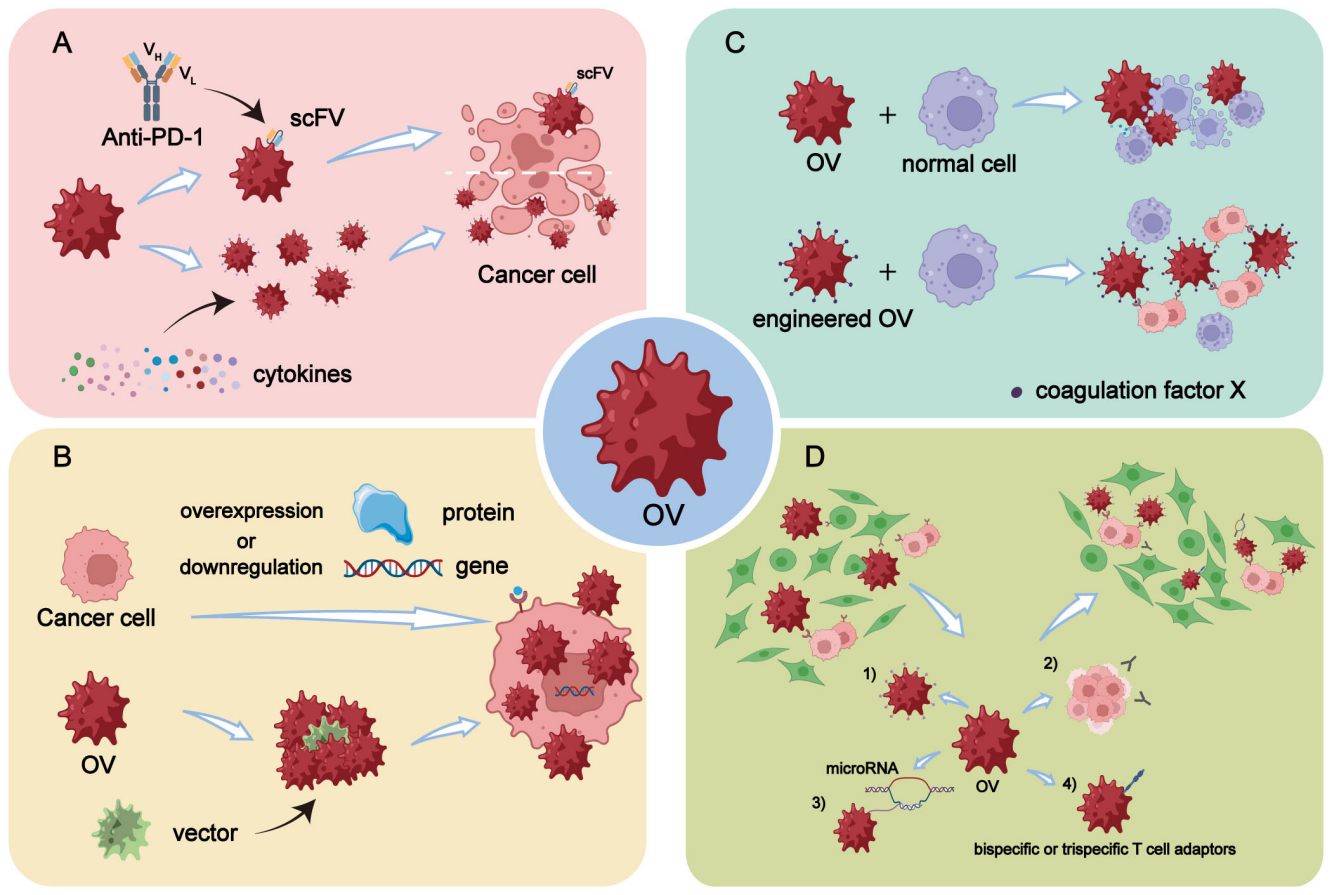
Engineering modifications of OVs. **(A)** Enhancement of *in vivo* immune response by arming OVs with scFV targeting PD-1 or by overexpressing specific cytokines through genetic engineering. **(B)** Enhancement of replication efficiency of OVs in tumor cells through genetic engineering by overexpressing or downregulating certain genes or proteins in tumor cells, or by selecting and designing more efficient virus vectors. **(C)** OVs are engineered to reduce off-target effects and damage to normal cells, making it a safer and more high-fidelity attenuated virus, thereby improving the safety profile of OV therapy. **(D)** Enhancement of tumor targeting of OVs through five main modification strategies: 1) Increasing virus affinity and binding activity to receptors overexpressed on tumor surfaces. 2) Utilizing differentiation of tumor cells to improve targeting accuracy. 3) Incorporating differentially expressed microRNAs into OVs through transgenic technology. 4) Arming OVs with bispecific or trispecific T cell engagers.

The immune checkpoint molecules play a crucial role in modulating the immune system. They function as co-stimulatory receptors present on various immune cells, transmitting inhibitory signals (18). Among the extensively studied immune checkpoint molecules are CTLA-4, TIM-3, and PD-1, which effectively regulate the immune response to prevent excessive immunological damage (19). A study conducted by Ju et al. demonstrated that OVs armed with a single-stranded fragment variable (scFv) targeting PD-1 effectively enhanced effector T cell activity in genetically engineered mice. The reported findings revealed that OVs expressing PD-1 inhibitors synergistically acted with anti-CTLA-4 or anti-TIM-3 agents to potentiate the immune response *in vivo* and consequently achieve tumor control (20) (**Figure 1A**).

#### 2.1.2 Engineered OVs expressing cytokines

Genetic engineering of OVs to express specific cytokines is an effective strategy to improve the immune response of OV. A range of antitumor responses can be regulated by cytokines (21), including (1): interferons (IFNs): IFNα, IFNβ, IFNγ (2); Interleukin (ILs): IL-2, IL-12, IL-15, IL-17, IL-18 (3); chemokines: CXCL9, CXCL10 and CCL5 (4); Granulocyte-macrophage colony-stimulating factor (GM-CSF) (5); Tumor necrosis factor (TNF) (**Figure 1A**).

##### 2.1.2.1 IFNs

IFN is divided into type I (IFNα and IFNβ) and type II (IFNγ), of which type II is mainly secreted by immune cells: T-helper (Th) 1 cells, natural killer cells (NK cells), etc. Expression of IFN in OVs can effectively induce tumor cell death through modulation of various pathways (22). Studies have demonstrated that oncolytic adenovirus (OAd) expressing IFN (IFN-OAd) significantly suppresses tumor growth in hamster pancreatic cancer models, leading to increased infiltration of tumor-infiltrating lymphocytes (TILs) (23). Furthermore, the incorporation of CD47 and IFNγ genes into MYXV results in the production of MYXV_IFNγ and MYXV_CD47, respectively. This dual expression strategy enhances anti-tumor immunity in a mouse melanoma model, highlighting the synergistic effects between CD47 and IFN (24). Therefore, it is evident that direct activation of immune cells by IFNs can potentiate *in vivo* immune responses.

##### 2.1.2.2 ILs

ILs are a class of small molecule proteins, possess the ability to both promote tumor cell growth and inhibit tumors in cancer (25). Due to their crucial role in tumor development, ILs can be incorporated into OVs for their antitumor functions. IL-2 functions as an anticancer cytokine by augmenting the activity of NK cells and cytotoxic T cells. Previous studies have demonstrated that IL-2 can be expressed in OVs such as VV, Sendai virus, Ad, and other vectors. Alternatively, IL-2 can be co-expressed with other anticancer transgenes in OVs to further enhance its immune characteristics (26). Recently, scientists developed an OAd that co-encodes TNFα and IL-2 and locally expresses them in hamster pancreatic cancer models. This approach modulates the TME by upregulating AIM2 expression and inhibiting tumor growth (27). IL-12 activates NK cells and T cells while promoting a Th1 type immune response. Previous studies have shown that engineering OAd (Ad5-ZD55-CCL5-IL12), which co-express CCL5 and IL-12, effectively increased the infiltration of chimeric antigen receptor (CAR)-T cells and TILs within tumors, resulting in potent anti-tumor effects with enhanced safety profiles (28). Additionally, treatment of colon cancer using oncolytic herpes simplex virus (oHSV) (O-HSV1211) modified to express both IL-12 and CXCL11 leads to increased infiltration of CD8^+^ T and CD4^+^ T cells into the tumor site (29). IL-15 functions as an upstream regulator of tumor-infiltrating CD8^+^ T cells, and the IL-15/IL-15Rα^+^ axis plays a crucial role in anti-tumor immunity (30). The researchers engineered a fusion protein combining IL-15 and IL-15Rα (designated OV-IL15C), which was expressed within gliomas and demonstrated the ability to enhance cytotoxicity against glioblastoma (GBM) both *in vivo* and *in vitro*, while also improving the survival of NK and CD8^+^ T cells (31). Furthermore, expression of IL-15 within oncolytic VV (32) as well as a novel OV (SG400-E2F/IL-15) (33) also resulted in enhanced immune response and antitumor activity *in vivo*. The production of IL-18 is observed in various cell types, including activated monocytes, macrophages, and dendritic cells (DCs). IL-18 plays a crucial role as a cytokine in cancer (34). Recombinant Pseudorabies viruses (PRVs), namely rPRV-PH20 and RPRV-IL-18-gamma-PH20, were engineered using pseudorabies virus (PRV) as the vector. The results demonstrated a significant increase in the infiltration of CD4^+^ T and CD8^+^ T cells within tumor cells infected with recombinant PRV strains. Moreover, the oncolytic effect was superior in the treatment groups receiving rPRV-IL-18-gamma-PH20, rPRV-PH20 alone or RPRV-IL-18-gamma-PH20 compared to the control group. Notably, among these groups, the best anti-tumor effect was observed with rPRV-IL-18-γ-PH20 treatment. Overall, co-expression of PH20 with IL-18 and IFNγ enhanced systemic anti-tumor immunity mediated by IL-18 (35).

##### 2.1.2.3 Chemokines

Chemokines are a subfamily of cytokines, produced by various cells in response to stimuli such as pathogens, drugs, or physical damage. These cells include white blood cells, endothelial cells, fibroblasts, and others. Chemokines play a crucial role in promoting cell migration throughout the body, particularly for white blood cells. They also have significant involvement in immune function regulation (36). The engineered expression of CCL5 shows promise as a method to enhance the immune response to OVs. For example, an OV called OV-Cmab-CCL5 was engineered to express CCL5 specifically within the TME. In GBM infected with OV-Cmab-CCL5, there was an increase in NK cell activity, T cell activity, and macrophage activity along with a decrease in tumor size (37). Other studies aiming to improve the immune response of OVs through engineering involve arming OAds with CXCL11 (38), overexpressing CXCR7 and CXCR4 in breast cancer cells using an armed OAd carrying CXCL12 (39), and utilizing CXCL10 as an armament for OAds (40).

##### 2.1.2.4 GM-CSF

The incorporation of GM-CSF into OVs has demonstrated significant benefits for cancer patients. Examples of OVs utilized include, but are not limited to, oncolytic vesicular stomatitis virus (VSV) (41), oncolytic VV (42), oHSV type 1 (oHSV-1) (43), OAd (44), oncolytic Herpesvirus talimogene laherparepvec (T-VEC) (45), and oncolytic reovirus (46). Additionally, the use of ONCOS-102 encoding GM-CSF and ONCOS-204 encoding ICOSL (the ligand of inducible T-cell co-stimulator) in modified adenoviruses further enhances the function of bi-specific antibodies (BsAbs)-activated T cells within melanoma cells. Notably, the combination of ONCOS-204 and EGFRxCD3 BsAb exhibits superior ability in augmenting T cell activation and cytotoxicity compared to ONCOS-102, with ONCOS-204 particularly significantly influencing CD4+ T cell subpopulations infected with tumor cells (44).

### 2.2 Improve the replication efficiency of OVs in tumor cells

The enhancement of OV replication efficiency in tumor cells can be approached from two perspectives (1): Manipulation of gene or protein expression levels in tumor cells through genetic engineering techniques (2). Selection and design of more efficient viral vectors (**Figure 1B**).

Through genetic engineering, certain genes and proteins can be manipulated to either increase or decrease their expression levels in tumor cells. This modulation of gene expression can synergistically enhance the replication efficiency and therapeutic efficacy of OVs. For instance, in an experiment, the death domain associated protein was down-regulated, leading to increased viral replication efficiency. Additionally, overexpression of the precursor terminal protein helped overcome poor viral replication and resulted in a higher production of total viral particles (47). To address the replication defect caused by insufficient arginine succinate synthetase 1 (ASS1) expression in tumors, a series of recombinant oncolytic MYXV constructs expressing exogenous ASS1 were generated (48). Moreover, upregulation of MHC class I chain-related polypeptide A (MICA), which serves as a ligand activating NK group 2D (NKG2D) receptor on NK cell and T cell subpopulations as an OV gene engineered transgene, was observed in tumor cells. The use of MICA-expressing oncogenic adenovirus named ICOVIR15KK-MICAMut demonstrated improved control over tumor growth compared to other viruses without MICA expression. This enhanced control is attributed to the virus’s increased replication efficiency within the tumor cells, leading to a higher oncolytic activity and more robust immune-mediated tumor cell destruction (49).

Through the screening and optimization of virus strains, more efficient and tumor-selective OVs can be identified. In a clinical trial for cancer treatment using reovirus serotype 3 Dearing (T3D), the Patrick Lee laboratory strain (T3DPL type) demonstrated enhanced replication efficiency and higher oncolytic performance (50). To enhance the anti-tumor immune activity of chimeric poxvirus deVV5, a chimeric virus with thymidine kinase deletion and a suicide gene, FCU1, was generated. The deVV5-fcu1 group exhibited superior replication efficiency compared to the control group, with results indicating that it achieved the highest rate of virus production from Hep G2 liver cancer cells (51). In a study involving engineered adenoviruses, an Ad5/3 serotype chimeric vector OV was designed utilizing adenovirus type 3 (Ad3) receptors. Findings revealed that the Ad5-ΔE3-Luc group displayed greater *in vivo* replication capacity than the Ad5/3-ΔE3-Luc group. These studies have shown that modifying OAd type 3 can improve the replication efficiency of serotype chimeric Ad5/3 vectors, which should be considered in future research endeavors (52). In a recent study, expression of a new generation OAd called Ad5 KT-E1A-IL-15 (TS-2021), along with Ki67 and TGF-β2 proteins, was generated to enable selective replication in GBM cells and enhance efficacy in killing GBM tumors (53).

### 2.3 Enhanced safety

Since the acceptance of viruses as the cause of pathogenicity has long been widespread, the safety of OVs has also been subject to conservative debate. It has been demonstrated that OVs kill tumor cells while inadvertently attacking normal cells, akin to the side effects observed with chemotherapy (54). In response to this concern, numerous studies have shown that OVs can be engineered into attenuated viruses with enhanced targeted specificity. For instance, deletion of the γ34.5 gene prevents oHSV-1, an OV, from infecting normal neurons (55–57). The OV-containing VG161 developed by Virogin Biotech Canada Ltd. helps maintain target sensitivity to drugs like acyclovir, effectively enabling control over its virulence and safety in clinical applications—an important advantage in terms of safety (58–60). Additionally, Yiye Zhong et al. have designed an OV containing targets for neuron-specific microRNA-124 and granulocyte-macrophage colony-stimulating factor, significantly enhancing its neuronal safety while minimally impacting its replication capacity (61).

Despite the tumor cell-specific engineering, there is a potential for off-target effects and unintended toxicities resulting from genetic manipulation. Additionally, issues such as viral mutation, evolution, recombination, cytotoxic gene products, and viral transmissibility may arise (62). Based on these findings, several studies have identified certain substances that can mitigate these risks when combined with OVs. For example, Alba et al. discovered that using Ad5-hexon in conjunction with coagulation factor X (FX) facilitates liver transduction (**Figure 1C**). They also developed a genetically FX-bound ablative Ad5-hexon vector for symptom relief purposes (63).

### 2.4 Improve targeting

The ability of OVs to specifically infect tumor cells while sparing normal cells is considered a promising approach for the safe and effective treatment of cancer (64). Despite numerous clinical trials confirming the excellent targeting capability of OV therapy, there still exist certain limitations that require resolution. There are four primary modification strategies available to enhance the tumor-targeting potential of OV (**Figure 1D**).

The first approach is to enhance the affinity and binding activity of the virus towards the overexpressed receptor on the tumor surface. By engineering OVs to specifically recognize receptors that are upregulated in tumors, their targeting can be improved. For instance, Yang et al. demonstrated that a chimeric adenovirus composed of Ad35 knobs and axles binding to Ad5 enhances targeting and oncolytic effects across various cancers by utilizing CD46 as a differential receptor (65). On the other hand, knowledge of membrane-associated tumor-associated antigens (TAAs) enabled researchers to fully engineer a virulent OV with selective tropism for tumor cells by substituting the viral glycoproteins involved in cell entry with antibody fragments targeting specific TAAs, such as HER2, PSMA, and MSLN (66, 67). Tomer Granot et al. employed Sindbis virus (SV) vectors to deliver TAAs and enhance viral targeting. They found that SV/TAA therapy’s efficacy stemmed not from direct tumor cell targeting, but from the transient expression of TAAs in lymph nodes draining the injection site. This mechanism prompted early T-cell activation, followed by a significant influx of NKG2D-expressing, antigen-specific cytotoxic CD8 T cells into the tumor. Ultimately, this led to the formation of long-lasting memory T cells, which conferred protection against rechallenge with tumor cells (68). Additionally, certain CD molecules that are overexpressed in malignant tumors serve as valuable targets for constructing targeted viral vectors to facilitate OV homing. For example, CD20-positive non-Hodgkin lymphoma (NHL) has been utilized to develop CD20-targeted MV vectors for lymphoma targeting with promising results (69). The increasing identification of tumor-specific receptors or antigens will provide more precise strategies for enhancing OV targeting.

Second, the targeting accuracy can be enhanced by leveraging the unique characteristics of tumor cells. For instance, OV can enhance its targeting selectivity by modulating genes or signaling pathways in tumor cells. Chen et al. achieved this by inhibiting the antiviral response of cells through blocking the alpha subunit of the IFN receptor using B18R (70). Additionally, overexpressing specific genes or proteins in tumor cells can also improve OV’s targeting selectivity. By replacing the endogenous E1A promoter with GOLPH2 (also known as GP73), E1B 55kD Ad deletion induces significant cytotoxic effects in prostate cancer stem cell (CSC)-like cells through GP73 overexpression and exhibits stronger oncolytic effects (71). Furthermore, armed with a full-length antibody against CD47, oHSV is capable of specifically targeting GBM and ovarian cancer (72, 73). Moreover, IL-12-carrying oHSV significantly elevates IL-12 levels within TME and the stimulates infiltration of effector T cells, NK cells, and APC into tumors to enhance anti-tumor efficacy (74).

Additionally, the integration of differentially expressed microRNAs into OVs through transgenic technology represents an alternative strategy to enhance targeting selectivity. In other words, OVs can be utilized as carriers to specifically deliver microRNAs for regulating cancer occurrence (75). MicroRNAs, which are short non-coding RNAs, play a crucial role in modulating gene expression by interfering with the translation of target mRNAs. Dysregulation of microRNAs has been implicated in tumor progression, invasion, angiogenesis, and metastasis across various types of cancer (76, 77). The OV vector demonstrates effective delivery of pre-tumor inhibition interference miRNA into tumor cells. Specifically, the interfering precursor microRNAs dissociate within the cytoplasm and undergo cleavage to generate mature microRNAs that subsequently lead to target mRNA inactivation. OAd carrying the tumor suppressor gene miR-143 induces apoptosis and reduces tumor growth by decreasing KRAS expression in HCT116 xenografts (78). Similarly, when oncolytic vesicular stomatitis virus serves as the carrier, miR-143 exhibits comparable antitumor effects in osteosarcoma cells (79). To further enhance oncolytic specificity while minimizing toxicity levels, Yang et al. have inserted miR-34a targets into both 5’ untranslated region (UTR) and 3’ UTR of the virus genome to develop a dual-targeting oncolytic Coxsackievirus B3 engineered variant that retains nearly complete oncolytic activity but with reduced toxicity levels (80).

Finally, the utilization of bispecific or trispecific T cell adaptors (BiTE or TriTE) molecules represents an alternative strategy for modifying OVs. BiTE is a recombinant protein consisting of two scFvs that bind to a T cell surface molecule and a malignant cell antigen, respectively, and arming OVs with a BiTE overcomes their extremely short serum half-life, while the next-generation TriTE includes three domains, such as CD3 × dual tumor antigens or tumor antigen × CD3/CD28.This technique involves linking two distinct ScFV antibodies, enabling each fragment to bind to both the surface of T cells and malignant cells. Consequently, this approach reduces the potential for immune escape due to antigen loss and minimizes side effects associated with targeted detumescence, ultimately improving tumor selectivity (81). Chen et al. demonstrated that CS1-NKG2D bispecial antibodies facilitate the augmentation of immune synapses between CS1^+^ multiple myeloma (MM) cells and NKG2D^+^ cytolytic innate as well as antigen-specific effector cells. As a result, these immune cells are activated, leading to improved clearance of multiple myeloma (82). Several other OVs carrying bifspecificity antibodies have exhibited distal effects through T-cell-mediated activation and tumolysis. Moreover, FAP and EGFR have been shown to enhance T cell activation and accumulation at the tumor site, thereby increasing anti-tumor efficacy (83–85). Furthermore, OV-encoded bispecific antibodies also promote T cell infiltration into the TME while exhibiting antitumor activity by enhancing T cell activation and cytokine production. Immune cold tumors, characterized by a lack of immune cell infiltration and activity, are typically resistant to immunotherapies. By promoting T cell infiltration and activation, OV-encoded bispecific antibodies help convert these immune cold tumors into immune hot tumors, which have a higher presence of active immune cells and are more responsive to immunotherapeutic interventions (86, 87).

## 3 Combination therapy

### 3.1 OV combined with chemotherapy

Chemotherapy, as a primary modality for cancer treatment, induces DNA damage by inhibiting processes such as DNA synthesis, mitosis, and cell division. Recent clinical trials have demonstrated the potential synergistic effect of combining chemotherapy with OVs, offering a promising alternative strategy in cancer therapy.

**Figure 2:**
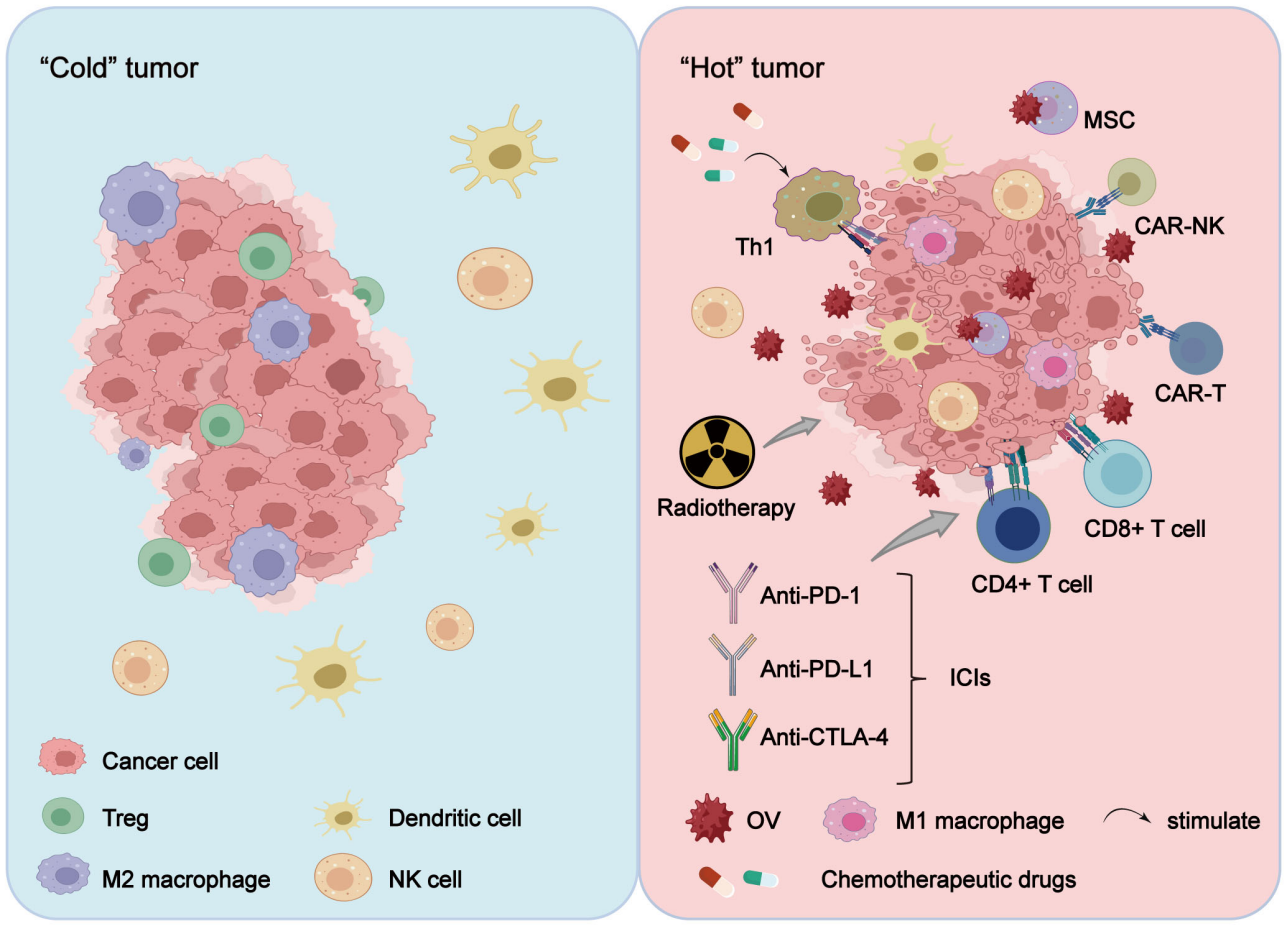
OV combination therapy reshapes the TME. Treatment of tumors with OVs alone or in combination with radiotherapy, chemotherapy, cell therapy and ICIs alters the tumor immune microenvironment, transitioning it from a “cold tumor” to a “hot tumor,” with the reshaping effect more pronounced in combination therapy. Furthermore, OV combination therapy reduces the tumor infiltration of immunosuppressive cells (including Treg cells and M2-polarized macrophages), while enhancing the proliferation of activated immune cells (such as CAR-T cells, CAR-NK cells, TILs, NK cells, M1-polarized macrophages, and DCs), thereby exerting a stronger anti-tumor effect.

Cyclophosphamide (CTX) is an alkylating chemotherapeutic agent and was the pioneering drug to be combined with OVs. CTX undergoes metabolic conversion into cytotoxic substances within tumor cells, thereby inducing tumor cell death. Moreover, it also functions as an immunosuppressive agent, impacting both innate and adaptive immunity in the body. Research has demonstrated that early-stage low-dose CTX combined with OAd therapy can induce TH-1 immunity by reducing regulatory T cells (Treg cells), transforming the TME from a “cold” state to a “hot” state, and enhancing anti-tumor efficacy (88) (**Figure 2**). Talimogene laherparepvec (T-VEC) is an oncolytic virus hypothesized to enhance the response of triple-negative breast cancer (TNBC) to neoadjuvant chemotherapy (NAC). The rationale for combining T-VEC with chemotherapy stems from the observation that TNBC tumors with significant pre-existing lymphocytic infiltration respond more favorably to neoadjuvant therapy. Preclinical studies have shown a synergistic effect between oncolytic viruses and chemotherapy, further supporting this combination approach. In a Phase II clinical trial of T-VEC combined with NAC in TNBC, this combination therapy was found to improve the response rate of TNBC tumors injected with T-VEC during NAC. This provides a theoretical foundation for further investigation of T-VEC plus NAC for TNBC treatment (89). Temozolomide (TMZ), another alkylating agent and immunomodulator, is extensively employed in treating various solid tumors such as glioma and melanoma. TMZ has been shown to enhance replication and tumor lysis effects of OAds in lung cancer cell lines but not non-cancerous cells; this augmented antitumor activity may partly result from autophagy induction in these lung cancer cells (90). Additionally, gemcitabine (GCB), a nucleoside analogue antimetabolite antitumor agent, is widely used alone or in combination with other anticancer agents across multiple cancers (91). In one study, researchers utilized replicative adenovirus-mediated double suicide gene therapy(Ad 5-DS) alongside standard intravenous GCB at recommended dosage levels; this approach proved safe and well tolerated among patients (92). These studies indicate the paramount importance of comprehending the interplay between OVs and anti-tumor chemotherapy drugs in advancing the development of combination therapy for cancer treatment.

However, subsequent studies have revealed that chemotherapy may exert a detrimental impact on the efficacy of oHSV immunoviral therapy. TMZ chemotherapy currently represents the standard treatment for GBM; however, when TMZ is combined with G47Δ-IL 12 to treat *in situ* tumor-bearing mice, it nullifies the beneficial effects of G47Δ-IL 12 and adversely affects intratumor T cells, macrophages, and spleen cells (93). Meanwhile, there remains limited clinical evidence supporting the combination of OV and chemotherapy; thus further experiments and clinical investigations are warranted to validate its effectiveness and safety.

### 3.2 OV combined with radiotherapy

Radiotherapy is the most efficacious cytotoxic modality for localized solid tumors (94). Its fundamental principle lies in irradiating the DNA of tumor cells, inducing DNA damage and impeding their indefinite proliferation until demise. It primarily encompasses alpha, beta, gamma rays, as well as diverse forms of X-rays. Radiotherapy is frequently employed in conjunction with chemotherapy to enhance patient survival (95). Nevertheless, the potent adverse effects of chemoradiotherapy and the heterogeneity of therapeutic efficacy are compelling researchers to explore novel combination therapies.

The combination of OV and radiotherapy not only exhibits a synergistic effect but has also demonstrated improved therapeutic efficacy in numerous studies. GBM, the most prevalent primary malignant brain tumor (96), is considered a “cold tumor” in immunology due to limited lymphocyte infiltration and unresponsiveness to current immunotherapies. Therefore, researchers are actively exploring novel techniques to convert “cold” tumors into “hot” tumors, thereby paving new avenues for cancer treatment (97) (**Figure 2**). One study demonstrated that GBM mice treated solely with OVs achieved a curative rate of 13.3 percent, while those treated with radiation alone had a curative rate of 21.4 percent. However, when the two therapies were combined, mice with brain cancer exhibited an impressive curative rate of up to 66.7 percent. The efficacy of combination therapy is further highlighted by the prolonged survival time observed in these mice. The median survival time for the control group (PBS group) was only 29 days, which increased to 39.5 days in the radiotherapy group and 41 days in the viral therapy group. Remarkably, when radiotherapy was combined with viral therapy, the median survival time exceeded 76 days. Further investigation revealed that this remarkable effect of combination therapy can be attributed to its significant increase in CD3^+^ cell count and proportion of CD3^+^ T/CD8^+^ T and CD8^+^ T/Treg cells in mice (98). Additionally, combining OVs with radiotherapy may enhance the “distant site effect” of radiotherapy (regression of unirradiated metastases at a distance from the irradiated site) (99), possibly due to radiotherapy’s ability to promote OV replication and increase cancer cell vulnerability (100). Moreover, OVs can augment immune checkpoint inhibitors’ effectiveness through interaction with radiotherapy. A study involving NDV demonstrated that combining NDV with radiotherapy and PD-1 antibody resulted in prolonged mouse survival and significantly inhibited tumor growth compared to groups treated solely with PD-1 antibody or combinations of PD-1 antibody/NDV or NDV/radiotherapy (101).

The current research on the combination of OV and radiotherapy is limited. However, existing studies have demonstrated significant potential in this combined therapy, which is expected to enhance the efficacy and safety of tumor treatment, offering hope to more patients.

### 3.3 OV combined with cell therapy

#### 3.3.1 OV combined with CAR-T

In recent years, the application of CAR-T cell therapy has demonstrated remarkable efficacy in cases where conventional cancer treatments are ineffective, particularly for untreatable blood system cancers such as leukemia, myeloma, and non-Hodgkin B-cell lymphoma (102). This approach selectively targets and eliminates tumor cells, leading to significant breakthroughs. Furthermore, there have been increasing clinical trials utilizing CAR-T cells for the treatment of solid tumors, with notable progress achieved in certain types of solid tumors. For instance, GBM exhibits high expression levels of IL-13Rα2 while normal brain cells show lower expression levels. This characteristic makes IL-13Rα2 a promising target for CAR-T cell therapy against GBM cancer. Brown et al. (NCT02208362) administered multiple infusions of IL-13Rα2-CAR-T cells directly into the resected tumor cavity via intracranial administration and observed regression of all intracranial and spinal tumors lasting 7.5 months (103). Additionally, a Phase I clinical study (NCT03182816) demonstrated the safety and feasibility of treating patients with advanced relapsed/refractory non-small cell lung cancer (NSCLC) using epidermal growth factor receptor (EGFR) CAR-T cells produced by the piggyBac transposon system instead of viral systems (104). However, despite these advancements in CAR-T cell therapy for solid tumors, several challenges and complications still exist including tumor heterogeneity, antigen escape by tumor cells, transportation limitations faced by CAR-T cells at the tumor site as well as invasion and expansion difficulties within the TME itself (105).

Notably, the combination strategy of OVs with CAR-T cell therapy holds great promise for enhancing the efficacy of CAR-T cells in solid tumors and overcoming associated challenges. Currently, there are four OVs approved worldwide for cancer treatment, among which T-VEC is the only OV approved by the Food and Drug Administration (FDA) that has demonstrated favorable safety and efficacy in clinical trials (100). Furthermore, successful CAR-T cell products already exist in the market, providing a strong foundation for combining OV and CAR-T therapy. Additionally, OVs have the ability to transform a “cold” tumor into a “hot” one. In a “cold” tumor, immunosuppressive cells like Treg cells and M2-polarized macrophages infiltrate surrounding tissues extensively while immune cell infiltration is insufficient and their function is suppressed. This allows tumor cells to evade attacks from the immune system. Conversely, in a “hot” tumor characterized by abundant infiltration of active immune cells associated with high response rates to immunotherapy (81), OVs can reshape such an environment effectively (**Figure 2**).

Secondly, numerous preclinical studies have demonstrated various enhancement effects achieved through combining CAR-T cells with OVs. For instance (1): Enhanced transport and persistence of CAR-T cells: Scientists infected DS CAR-T cells with VSV and reovirus as delivery vehicles to target tumors; these OVs replicated within tumor cells leading to expansion of DS CAR-T cell population while causing rupture of tumor cells. Moreover, systemic stimulation by reovirus reactivated virus-specific CAR-T cells resulting in long-term remission lasting over 60 days in six out of seven mice tested; this approach also increased *in vivo* persistence of CAR-T-cells significantly (106) (2). As previously mentioned, OVs armed with multiple cytokines or chemokines have been engineered to enhance the therapeutic efficacy of CAR-T cell therapy. These include TNFα (107), IL-21 (108), IL-7 (109), CXCL11 (110), among others. Genetically modified OVs can produce a broader range of chemokines that promote the infiltration of cytotoxic T cells, DCs, macrophages, and other immune cells into the TME for improved anti-tumor effects (111). Wang et al. evaluated the use of CXCL11-armed OAds to augment CAR-T cell infiltration in GL261 GBM models and reprogram the immunosuppressive TME. This approach resulted in increased infiltration of CD8^+^ T lymphocytes, NK cells, and M1-polarized macrophages while reducing myeloid suppressor cells (MDSCs), Tregs, and M2-polarized macrophages. The study demonstrated that combining CXCL11 with oAd within the tumor environment led to a sustained anti-tumor response (38) (3). OV-mediated targeted delivery of surface antigens in tumor cells (112). Anthony K Park and colleagues developed an oncolytic VV that expresses a non-signal-intercepting CD19 protein (CD19t), enabling targeted delivery of CD19t to the surface of solid tumor cells. This viral infection induces antigen-specific CD19-CAR-T cell-mediated antitumor activity, leading to both viral release from dying tumor cells and expansion of CD19t expression in the tumor (113). Additionally, an oHSV (oHSV T3011) was engineered to deliver truncated CD19 and BCMA double antigens in combination with either CD19-specific CAR-T (CAR-T^CD19^) or BCMA-specific CAR-T (CAR-T^BCMA^) cell therapy, resulting in a synergistic antitumor response. oHSV T3011 is a recombinant herpes OV expressing IL-12 and anti-PD-1 antibodies, which helps improve the inhibitory TME and enhances overall anti-tumor activity (114).

It is worth noting that there exist antagonistic mechanisms in the combination therapy of OV and CAR-T. The VSV-IFNβ induces the release of type I interferon, which subsequently up-regulates inhibitory receptors LAG3, PD-1, and TIM3. This effect is particularly pronounced in transduced cells and correlates with the expression level of CAR. Therefore, when used in conjunction with CAR-T therapy, IFNβ may impede the antitumor activity of CAR-T cells by stimulating the CAR signaling pathway to enhance CAR expression and modulating inhibitory receptor expression to restrict the active state of CAR-T cells (115).

#### 3.3.2 OV combined with CAR-NK

NK cells, an integral part of the innate immune system, possess a distinct cytotoxic mechanism compared to adaptive T lymphocytes and can directly eliminate target cells without prior antigen sensitization (116). NK cells express a diverse array of activating and inhibitory receptors that regulate their activity. Activating receptors include NCR, CD16, NKG2D, DNAM1, and signaling lymphocyte activation molecule (SLAM), while inhibitory receptors comprise immunoglobulin-like receptors (KIRs), NKG2A, and leukocyte immunoglobulin-like receptors (LILRs) (117, 118). These receptor families play a crucial role in modulating the immune response of NK cells towards tumors. The emergence of CAR technology has demonstrated significant potential in cancer immunotherapy by enhancing the recognition and elimination capabilities of immune cells (102, 119). Currently, there are five CAR-T cell therapies approved by the U.S. FDA for treating B-cell-derived lymphoma, leukemia, as well as hematological malignancies such as multiple myeloma (120). However, due to the limitations of CAR-T cells in the treatment of solid tumors, such as their inability to infiltrate tumor tissue, lack of suitable targets, and associated toxicity (102), it is imperative to discover novel strategies and technical approaches to overcome these challenges and enhance the efficacy and feasibility of CAR-T cell therapy for solid tumors. CAR-NK cell therapy may serve as a promising alternative. In contrast to CAR-T cell therapy, NK cells can be derived from various sources including peripheral blood, umbilical cord blood, induced pluripotent stem cells, and NK cell lines (121). Therefore, CAR-NK cells can be produced on a large scale with significantly reduced treatment time. Moreover, unlike CAR-T cells, CAR-NK cells are not restricted by histocompatibility complex (MHC) on the surface of target cells and exhibit a broader spectrum of anti-tumor effects. A Phase 1 and 2 clinical trial (NCT03056339) involving the injection of CD19 CAR-NK cells into 11 patients with relapsed or refractory CD19-positive cancers demonstrated that this therapy was effective in most patients without any apparent secretion of inflammatory cytokines such as IL-1 or IL-6. Furthermore, no association was observed between this therapy and the development of cytokine release syndrome, neurotoxicity or graft-versus-host disease (122).

Based on the remarkable efficacy of combination therapy using CAR-T cells and OVs, researchers have proposed combining OVs with CAR-NK cells. As tumor cells infected with OVs dissolve and rupture, they express ligands related to cell stress such as MICA/B proteins and ULBP family proteins, thereby increasing the recognition targets for CAR-NK cells. This leads to more effective removal of residual tumor cells and a more comprehensive clearance effect (119). Xilin Chen et al. found that EGFR was highly expressed on the surface of breast cancer cells, and using EGFR-CAR-NK-92 cells alone or in combination with oHSV-1 resulted in significant killing of cancer cells. The combination produced a more effective killing effect than monotherapy and significantly extended survival time in tumor-bearing mice, making it an effective treatment for breast cancer brain metastases (123). Recently, multiple GBM cell lines infected with herpes simplex type I virus (OV-IL15C) expressing human IL-15/IL-15Rα sushi domain fusion protein secreted soluble IL-15/IL-15Ra complex to improve the survival rate of NK and CD8^+^ T cells. When combined with EGFR-CAR-NK cells, this increased persistence of CAR-NK cell activity synergistically suppressed tumor growth and significantly improved survival rates (31).

#### 3.3.3 OV combined with TILs

TILs typically comprise clusters of T cells and B cells (124, 125), and the type and persistence of these immune cells within the tumor are associated with the prognosis of cancer patients (124). The findings of studies have demonstrated that treatment of tumors with OVs had a favorable impact on both TIL infiltration and activity, thereby influencing tumor progression. The combination of OV with TIL may yield enduring antitumor effects by enhancing TIL activity. For instance, modified OVs based on OX40L and IL-12 represent a promising therapeutic approach for solid tumors. By infecting tumor cells, this particular OV can provide the necessary signals for activating T cells while transforming tumor cells into artificial antigen-presenting cells (126). Consequently, it not only induces T cell activation but also stimulates their cytotoxic function. Notably, significant tumor regression as well as long-term immune memory effects were observed when combined with TIL in tumor models (126). This suggests that this approach holds potential for persistent and effective control over solid tumor growth and metastasis. With a further comprehensive understanding of the relevant experiments, we can gain a deeper understanding of how OX40L and IL-12 based modified OVs mechanistically inhibit solid tumor growth while optimizing curative rates. Ultimately, this prospective strategy offers new hope in cancer management field as it could become an integral component of future personalized cancer management strategies (126). Another study demonstrated that the combination of OAd encoding human IL-2 (hIL2) and TNFα, along with TILs, exhibited prolonged efficacy, increased the frequency of both CD4 and CD8 TILs *in vivo*, and augmented splenocyte proliferation ex vivo, suggesting that the cytokines were important for T cell persistence and proliferation, significantly enhancing the effectiveness of TIL therapy (127). TNFα and IL-2 are incorporated into OAds to selectively infect cancer cells through tumor-specific promoters and knob protein exchange, thereby enhancing cancer cell entry (128). Moreover, utilizing TILs as carriers to deliver the virus to tumors can augment both the concentration and efficacy of the virus within the tumor site (128).

At the same time, OV can exert a stronger anti-tumor effect by increasing TIL infiltration and enhancing TIL function.

##### 3.3.3.1 Increased TIL infiltration

Engineered OVs have the potential to enhance the infiltration of TILs during disease treatment. Genetically modified herpetic virus type 1 (HSV-1) G207 has been utilized in pediatric patients with high-grade glioma for therapeutic purposes. By inducing an immune response and attracting cells through G207 infection, it is possible to convert “cold” tumors into “hot” tumors, thereby increasing the quantity of TILs and improving treatment efficacy (129) (**Figure 2**). In the context of GBM treatment using G47Δ, a third-generation oncolytic HSV-1 with triple mutations, a significant augmentation in CD4^+^ and CD8^+^ lymphocyte populations was observed as they rapidly infiltrated into tumor tissue. The sustained increase in these lymphocytes not only persisted over time but also exhibited a strong correlation with enhanced treatment outcomes (130). By genetically modifying the tumor-soluble bovine pox virus to express IL-7 and IL-12, it is possible to enhance the sensitivity of anti-PD-1 and CTLA4 antibody therapy. This modification also leads to an increase in the expression of major histocompatibility complex II (MHC II) in antigen-presenting cells, thereby altering the immune status and systemic immune response within the TME. Consequently, there is an augmented infiltration of CD8^+^ T cells, CD4^+^ T cells, NKT cells, and NK cells into the tumor site (131). The introduction of adenovirus-mediated n-terminal gasdermin domain expression induces pyroptosis in tumor cells while recruiting TILs into the brain. This process enhances their infiltration and subsequently improves anti-tumor efficacy (132). Delta-24-RGD OAd directly lyses tumor cells and activates anti-tumor immune responses, promoting invasion by T cells (133). OBP-502 is a telomerase-specific OAd that releases immunogenic cell death molecules such as adenosine triphosphate (ATP) and high mobility group box 1 protein (HMGB1) upon treatment. This release recruits CD8^+^ lymphocytes while inhibiting Foxp3 positive lymphocyte infiltration into tumors, resulting in antitumor effects (134). OVs modified with glycosylation -PEGX can improve selective infection and killing ability against tumor cells. Additionally, they enhance infiltration of T cells and NK cells, thus enhancing anti-tumor immune responses (135). Treatment with oncolytic HSV-1 results in regression of lymphoma-guided tumors accompanied by significant invasion of antigen-specific CD8^+^ T cells (136). In addition, MSC-mediated delivery of OAds to osteosarcoma leads to increased infiltration of TILs (137).

##### 3.3.3.2 Enhancement of TIL function

The use of OV or modified OV treatment for corresponding diseases may facilitate the augmentation of TIL activation, metabolic capacity, and durable anti-tumor response. Researchers have genetically engineered the OV to incorporate humanized PD-1 single-chain antibodies (hPD-1scFv) in order to enhance its impact on TILs (20). Modified OV therapy has demonstrated an enhanced anti-tumor effect on CD8^+^ T cells, leading to increased infiltration of effector CD8^+^ T cells into tumors and establishment of memory CD8^+^ T cells, while concurrently reducing associated depletion of CD8^+^ T cells (20). The expression of leptin by engineered OVs within tumor cells can promote metabolic reprogramming of TILs, thereby enhancing their metabolic activity and facilitating disease treatment (138). Recently, it has been reported that oHSV can reshape the immune microenvironment in pancreatic ductal adenocarcinoma (PDAC) by augmenting immune activity. Through utilization of scRNA-seq and multicolor fluorescence activated cell sorting analysis techniques, researchers observed a significant reduction in tumor-associated macrophages (TAMs), particularly anti-inflammatory macrophages, following oHSV treatment. Additionally, there was an increase in the proportion of TILs including activated cytotoxic CD8^+^ T cells and Th1 cells (139). Tumor cells infected with CCL5-modified OVs were able to produce CCL5 without compromising infectivity, thereby promoting NK cell accumulation and augmenting the therapeutic efficacy (140). Vv-scfv-tigitt, an engineered OV carrying ICIs, has been demonstrated to induce T cell infiltration and enhance CD8^+^ T cell activation in tumor models, leading to the establishment of long-term immunity (141). The CD40L-armed oncolytic HSV enhances the cytotoxicity of T cells and promotes the activation of DCs and T cells in the TME by inducing the expression of TAAs and enhancing the immunogenicity of tumor cells. This approach shows potential as a therapeutic strategy for PDAC (142). The OX40L-armed OV (OV-mOX40L) reduces the number of Foxp3^+^ Tregs and activates CD4^+^ and CD8^+^ T cells through interaction with OX40L. Additionally, it decreases exhausted CTLs while promoting t cell activation, leading to increased release of inflammatory cytokines such as IFNγ. Consequently, this transforms the immunosuppressive TME into a more immunologically active state (143). Combined treatment with an OV and anti-PD-1 significantly increases levels of CD8^+^ and CD4^+^ T cells, activates the central immune system, and enhances therapeutic efficacy (144). Adenoviruses have potential as an immunotherapy tool for stimulating TIL activity by delivering TNFα and IL-2. The results suggest that adenovirus can reshape cytokine responses and activate TILs in the TME, thereby improving their antitumor reactivity (145).

#### 3.3.4 MSCs are used as vectors to transport OVs

The utilization of OVs for disease treatment may elicit an immune response, thereby impeding viral spread and infection, consequently diminishing treatment efficacy. Moreover, due to the absence of specific targeting in virus administration, non-target tissues may be susceptible to infection, resulting in adverse reactions and toxicity. Simultaneously, pre-existing immune tolerance can hinder inter-tumoral migration of the virus, posing a challenge for treating metastatic diseases as both injected and distant tumors need to be targeted (146). To overcome these limitations associated with OV administration, researchers are actively engaged in a series of exploratory investigations.

MSCs possess low immunogenicity, inherent tumor tropism, multi-lineage differentiation potential, excellent migratory capacity (147), homing ability, and other therapeutic properties. These innate characteristics make them ideal candidates for drug delivery and OV vectors (148, 149). Utilizing MSCs as carriers of OVs for tumor therapy can enhance viral delivery efficiency, augment the antitumor effect of viruses on cancer cells, enable precise drug targeting, and mitigate systemic side effects (150).

The researchers improved the targeting ability of MSCs and modulated the drug release time to enhance the efficacy of OAds, enabling them to function as a factory and vector for OAds. They also evaluated tumor bioavailability after MSC injection. This approach significantly increased viral production, tumor targeting, timely viral release at the tumor site, and the antitumor efficacy of the oncolytic adenovirus. These findings indicate that engineered MSCs can substantially boost the antitumor effects of oncolytic viruses without compromising safety, potentially expanding the clinical applicability of oncolytic adenoviruses (151). In a mouse model of pulmonary melanoma, MSCs were utilized to deliver an IL-15-carrying tumor-lytic MYXV construct, resulting in sustained viral presence and increased infiltration of NK cells and CD8^+^ T cells. This approach transformed the tumor into a “hot tumor” and induced significant regression (152) (**Figure 2**). Another study encapsulated CF33 within NSCs to enhance its delivery in a cisplatin-resistant peritoneal ovarian metastasis model, providing a more efficient alternative compared to conventional delivery methods (153). The MYXV, carrying the LIGHT (TNFSF14) gene, was pre-loaded into adipose-derived mesenchymal stem cells (ADSCs) and utilized for the treatment of pancreatic cancer in mice. The findings demonstrated that when combined with carrier cells, the virus could be efficiently delivered to pancreatic cancer lesions, enabling cell survival while effectively eliminating pancreatic cancer cells. This resulted in tumor regression and prolonged survival time in treated mice (154). Furthermore, compared to traditional OV treatment for colorectal cancer, combination therapy employing MSCs as carriers and prodrug activation exhibited superior therapeutic efficacy and safety. It also possessed tumor specificity and innovative advantages through prodrug activation (155). Therefore, utilizing MSCs as carriers for transporting OVs presents a novel approach to tumor virotherapy with promising application prospects.

### 3.4 OV combined with ICIs

ICIs are a form of immunotherapy that has garnered significant attention in recent years for their potential in tumor treatment by targeting and inhibiting immune checkpoints, such as CTLA-4 and PD-1, to activate the immune response (156). However, studies have indicated that ICI may not be suitable for all patients, with some experiencing severe adverse reactions during treatment (157). Only a minority of patients achieve favorable disease control following ICI therapy. Furthermore, ICI showed no efficacy against immunologically “cold” tumors, characterized by low levels of TILs (158). Consequently, numerous researchers are actively exploring substances capable of inducing the conversion of “cold” tumors into “hot” tumors when used alongside ICI therapy to combat the disease.

OVs have been demonstrated in numerous studies to elicit anti-tumor immune responses, augment the efficacy of existing cancer therapies, and modulate unresponsive TME, thereby converting “cold” tumors into “hot” tumors and enhancing their sensitivity to checkpoint blockade immunotherapy (159) (**Figure 2**). Consequently, OVs serve as an ideal adjunct to ICIs. Sachin R Jhawar et al. investigated the effectiveness of this combination therapy using *in vitro* mouse models, human cancer cell lines, and murine skin cancer models. Following initial treatment with OV and radiotherapy, ICIs were subsequently administered to establish a triple therapy comprising OV, radiotherapy, and ICI. The results revealed that this triple therapy effectively suppressed tumor growth and prolonged survival. In addition, the researchers reported that a PD-1 refractory patient with squamous cell carcinoma of the skin received a longer period of disease control and survival after triple therapy with OV, radiotherapy, and ICI, and the tumor did not show significant progression for 44 months. The mechanism of the above results is that OV combined with radiotherapy and ICI, can not only transform immunologically “cold” tumors into “hot” tumors, but also improve the infiltration of CD8+ T cells (160). ONCOS-102 is a highly engineered Ad vector that has undergone extensive preclinical investigations in recent years (161) and has advanced to Phase I clinical trial stage (NCT03003676) which used in combination with the ICI pembrolizumab. The Phase I trial, which enrolled 12 patients with advanced or unresectable solid tumors, demonstrated that ONCOS-102 exhibited no dose-limiting toxicity and reached the maximum tolerated dose at the tested level, as compared to the pre-treatment dosage. Analysis of tumor biopsies following combination therapy revealed a significant increase in infiltration of CD3^+^ T cells (5.9-fold) and CD8^+^ T cells (4.0-fold). Among the 10 patients evaluated by PET/CT scans at 3 months, disease control was observed in 4 patients (40%), with a median overall survival of 9.3 months (162).

In addition to demonstrating efficacy, numerous studies have substantiated the safety of combining OVs with ICIs. In a study conducted by Targovax ASA et al., where ONCOS-102 was combined with pembrolizumab for treating PD-1-resistant advanced melanoma patients, treatment tolerance was well-established. Out of the 20 patients involved, objective response was achieved in seven cases along with regression of lesions at non-injection sites - indicating systemic antitumor effects resulting from local administration of ONCOS-102. Sequential biopsies performed on injected tumors showed substantial infiltration of CD8^+^ T cells and CD4^+^ T cells post-administration of ONCOS-102 injections. Therefore, these findings suggest that further investigation into the combination therapy involving ONCOS-102 and PD-1 inhibitors holds promise for PD-1-resistant melanoma treatment (163). Professor Gelareh Zadeh’s research team from the University of Toronto in Canada published their phase I/II clinical study results in Nature Medicine, showing that combining OV therapy DNX-2401 with pabolizumab for recurrent GBM treatment resulted in a 52.7% one-year survival rate, and some patients even survived after 60 months of treatment. Two patients achieved complete response (CR) and three patients achieved partial response (PR). With an ORR of 10.4%(90% CI:4.2-20.7%) in the intention-to-treat population and 11.9% in patients with the maximum trial dose (declared dose), this combination regimen is expected to become a novel treatment option for recurrent GBM (164). In addition, Hemminki’s team recently reported on two OVs expressing TNFα and IL-2, respectively. In melanoma experiments conducted on mice, they found that when combined with anti-PD-1 antibodies, the virus significantly increased CD8^+^ T cell numbers compared to using only the virus alone; furthermore, combining OV with ICIs significantly inhibited tumor development and prolonged survival time compared to using only the virus alone or ICIs alone. Interestingly, combining NDV with anti-CTLA-4 antibody also showed synergistic effects in mouse tumor models by increasing CD8^+^ T cell infiltration while inhibiting tumor growth and prolonging survival time (165). T-VEC is a genetically engineered oHSV-1 (166). In a single-center, single-arm, Phase II study, 24 resectable patients with stage IIIB-IVM1a melanoma who received intrafocal T-VEC injection and systemic nebuliuzumab had a major pathological complete response rate of up to 45%. The main mechanism is that the combination of T-VEC and ICI changes the infiltration of immune cells, transforming “cold” tumors into “hot” tumors, thus enhancing the immune response (167). In an interim report on another clinical trial that has begun studying C-REV in combination with the PD-1 inhibitor nivolumab (NCT03259425) in patients with resectable stage IIIB, IIIC, or IVM1a melanoma, Patients treated with the combination of C-REV and nivolumab showed higher T cell infiltration than patients treated alone in previous clinical trials (168).

In summary, the combined application of OV and ICIs has yielded remarkable results by enhancing lymphocyte infiltration and effectively prolonging survival. These findings strongly support the notion that OVs serve as ideal adjuvant therapies for ICIs.

### 3.5 OV Combined with ultrasound-targeted therapy

Ultrasound-targeted therapy is a method that uses the physical properties of ultrasound to precisely locate and treat tumors. Its main principle involves the cavitation and thermal effects of ultrasound to disrupt tumor tissue while using acoustic radiation force to enhance microbubble-mediated ultrasound-targeted drug delivery systems. This improves the concentration of drugs at the tumor site and enhances therapeutic efficacy. Additionally, ultrasound can temporarily increase the permeability of tumor vasculature, promoting the penetration of drugs or gene carriers, thereby further enhancing treatment efficiency (169). Due to its non-invasive nature, precise targeting, and low side effects, ultrasound-targeted therapy has shown great potential in treating various solid tumors (170–172).

The combination of ultrasound-targeted therapy with OVs opens new avenues for cancer treatment. OVs can selectively infect and kill tumor cells, while ultrasound-targeted technology can enhance the infection efficiency and distribution precision of OVs (173). For instance, Bazan-Peregrino et al. studied how ultrasound-induced cavitation improves the extravasation and distribution of a potent breast cancer-selective oncolytic adenovirus, AdEHE2F-Luc, to tumor areas distant from blood vessels. Inertial cavitation was found to be more effective than stable cavitation in enhancing the delivery, distribution, and efficacy of the oncolytic virus (174). Moreover, using microbubble carriers to load OVs and employing ultrasound-guided targeted delivery ensures efficient release and infection of OVs at the tumor site. Greco et al. demonstrated that ultrasound-targeted microbubbles/Ad.mda-7 (a replication-incompetent adenovirus expressing melanoma differentiation–associated gene-7/interleukin-24) significantly reduced tumor burden in xenografted nude mice. The microbubbles burst under ultrasound, releasing OVs directly into tumor cells and enhancing the oncolytic effect (175). Additionally, the mechanical action of ultrasound can increase the permeability of tumor cell membranes, enhancing OV entry and broader intratumoral spread. For example, Okunaga et al. found that ultrasound increased the efficiency of HSV-1 infection in human squamous cell carcinoma cells and tumors in nude mice, potentially enhancing the antitumor effect of oncolytic HSV-1 in head and neck cancer treatment (176).

This combined therapy strategy not only improves the targeting and therapeutic efficacy of OVs but also reduces their distribution in normal tissues, thereby minimizing adverse effects. Various targeting ligands incorporated into acoustically active materials, such as nanoparticles (170, 177), polymeric micelles, and liposomes (178), contribute to this effect. Therefore, the future application of ultrasound-targeted technology combined with OVs promises to become an efficient, precise, and comprehensive cancer treatment strategy, offering new hope for cancer patients.

## 4 Clinical trials

In recent years, OV genetic engineering therapy has demonstrated significant potential in the field of tumor treatment. Researchers are utilizing genetically modified viruses, such as MV and HSV, to develop precise methods for selectively eliminating tumor cells while preserving normal cells. We present a comprehensive overview of major clinical trials involving engineered OVs to explore their potential applications in oncology therapy (**Table 1**). For instance, an embryonic MV (MV-CEA) expressing recombinant carcinoembryonic antigen (CEA) and an oncolytic MV (MV-NIS) encoding a thyroid sodium-iodine cotransporter were employed in a clinical trial for ovarian and peritoneal carcinoma (NCT00408590). These studies aimed to assess the safety and optimal dosage of engineered viral therapy for progressive, recurrent, or refractory tumors. Another clinical trial focused on recurrent brain cancer (NCT00028158), where engineered herpesvirus G207 was directly injected into the brain and administered bedside after surgical removal to evaluate its safety, therapeutic efficacy, and novel treatment possibilities for patients with brain cancer. Additionally, recent clinical research has primarily focused on evaluating the safety and efficacy of the engineered oncolytic virus injection R130 (a modified HSV-1 containing the gene coding for anti-CD3 scFv/CD86/PD1/HSV2-US11) in patients with recurrent/refractory cervical and endometrial cancers (NCT05812677). In summary, these clinical trials underscore the potential of engineered OVs as a promising strategy in oncology, highlighting their safety, efficacy, and innovative therapeutic applications.

**Table 1:** Major clinical trials of genetically engineered OVs.

Start time	Engineered OVs	Enhancements and modifications in genetically engineered OVs	Cancer type	Purpose of the study	Phase	Status	Clinical trial number
2004	MV-NIS	oncolytic MV encoding thyroidal sodium iodide symporter	Ovarian cancer, primary peritoneal cavity cancer	Side effects and optimal dosage	I	Completed	NCT00408590
2001	G207	G207 has been modified from the herpes virus that causes cold sores (called herpes simplex virus type 1 or HSV-1)	Astrocytoma, glioblastoma	Safety and efficacy assessments	Ib/II	Completed	NCT00028158
2017	rQNestin34.5v.2	rQNestin34.5v.2 is a genetically engineered HSV-1 virus	Brain cancer (cancernaplastic oligodendroglioma of brain), astrocytoma	Safety assessment and determination of appropriate dose	I	Completed	NCT03152318
2013	MV-NIS	oncolytic MV encoding thyroidal sodium iodide symporter.	Head and neck squamous cell carcinoma, breast cancer stage IV	Side effects and optimal dosage	I	Completed	NCT01846091
2017	MV-NIS	oncolytic MV encoding thyroidal sodium iodide symporter.	Metastatic malignant peripheral nerve sheath tumor, recurrent malignant peripheral nerve sheath tumor	Side effects and optimal dosage	I	Recruiting	NCT02700230

MV-NIS, oncolytic measles virus encoding thyroidal sodium iodide.

At the same time, the clinical research on the combination of OVs with other drugs for tumor treatment has demonstrated a robust trend. These studies have investigated the feasibility and safety of combining OVs with immunotherapy drugs, ICIs, etc., aiming to enhance the efficacy of tumor treatment and potentially overcome resistance to conventional and immunotherapies (**Table 2**). For instance, a study (NCT02977156) aimed to assess the feasibility and safety of combining anti-CTLA-4 therapy with intratumoral injection of Pexa-Vec, an OV. This combination sought to improve antitumor effects by inducing virus-mediated tumor cell death and release of tumor antigens, as well as recruiting/maturing/activating antigen-presenting cells through GM-CSF induction while blocking/depleting Tregs via anti-CTLA-4. Furthermore, recent clinical trials have been initiated to explore the potential of OV combination therapies. The NCT06196671 trial aims to evaluate the efficacy of an oncolytic virus combined with a PD-1 inhibitor in patients with advanced pancreatic cancer. Additionally, the NCT06346808 trial is designed to explore the safety and efficacy of combining an oncolytic virus with a PD-1 inhibitor and chemotherapy as preoperative therapy for patients with borderline resectable and locally advanced pancreatic cancer. In summary, these clinical trials underscore the promising potential of OV combination therapies in enhancing tumor treatment efficacy and overcoming therapeutic resistance, particularly through the integration of ICI or chemotherapy strategies.

**Table 2:** Major clinical trials of OV combination therapy.

Start time	OVs	Combination drugs	Cancer type	Purpose of the study	Phase	Status	Clinical trial number
2017	Pexa-Vec	IT ipilimumab (anti-CTLA4 Ab)	Metastatic tumor, advanced tumor	Feasibility, safety and anti-tumor effects after combination therapy	I	Completed	NCT02977156
2021	OVV-01	pembrolizumab (anti-PD-1 monoclonal antibody) or atezolizumab	Neoplasms	Evaluation of safety, tolerability, and efficacy after combination therapy	I	Recruiting	NCT04787003
2019	OH2	HX008 (an anti-PD-1 antibody)	Gastrointestinal cancer, solid tumor	Evaluation of safety and efficacy after combination therapy	I/II	Recruiting	NCT03866525
2021	RT-01	Nivolumab (ICIs)	Advanced solid tumor	Evaluation of safety, tolerability and preliminary efficacy after combination therapy	I	Current recruitment status is unknown	NCT05122572
2012	CGTG-102	low-dose oral cyclophosphamide	Malignant solid tumor	Safety and recommended dose after combination therapy	I	Completed	NCT01598129
2013	DNX 2401	TMZ	Recurrent tumor, glioblastoma multiforme	Evaluation of safety, tolerability, and toxicity after combination therapy	I	Completed	NCT01956734
2017	Pexa-Vec (JX-594)	Tremelimumab	Colorectal neoplasms, colorectal cancer, refractory cancer	Evaluation of safety, tolerability and feasibility after combination therapy	I/II	Completed	NCT03206073
2022	H101	Camrelizumab (PD-1 inhibitors)	Bladder cancer	Safety and efficacy assessment after combination therapy	II	Recruiting	NCT05564897
2020	CAdVEC	HER2 specific CAR-T cells	Advanced HER2 positive solid tumors	Safety and efficacy assessment after receiving specific T cells after intratumoral CAdVEC injection	I	Recruiting	NCT03740256
2023	H101	PD-1 inhibitors	Advanced malignant pleural mesothelioma	The efficacy and safety of patients with malignant pleural mesothelioma resistant to advanced PD-1 inhibitors after combination therapy	Observational	Recruiting	NCT06031636
2012	GL-ONC 1	CDDP (radiation therapy and cisplatin)	Cancer of head and neck	Safety and tolerability after combination therapy	I	Completed	NCT01584284
2024	TILT-123	Pembrolizumab	Locally advanced, unresectable, refractory and/or metastatic solid tumors	Safety, tolerability, and preliminary antitumor efficacy after combination therapy	I/II	Recruiting	NCT06265025
2020	LOAd 703	Atezolizumab	Malignant melanoma	Evaluation of safety and efficacy after combination therapy	I/II	Completed	NCT04123470
	HF10	Ipilimumab	Malignant melanoma	Whether combination therapy is effective in patients with stage IIIB, IIIC, or stage IV unresectable or metastatic melanoma	II	Completed	NCT02272855
2006	MV-NIS	Cyclophosphamide	Recurrent plasma cell myeloma, refractory plasma cell myeloma	Side effects and optimal dose after combination therapy	I/II	Completed	NCT00450814
2017	NIS	Cyclophosphamide, Ipilimumab and nivolumab or cemiplimab	Multiple myeloma, acute myeloid leukemia and T-cell lymphoma	Optimal dose and side effects after combination therapy	I	Recruiting	NCT03017820

MV-NIS, oncolytic measles virus encoding thyroidal sodium iodide symporter; NIS, VSV-hIFNbeta-sodium iodide symporter; Pexa-Vec, pexastimogene devacirepvec; TMZ, Temozolomide; HER2, human epidermal growth factor receptor.

Taken collectively, these clinical studies unveil the potential of OV genetic engineering therapy in the treatment of tumors. By precisely targeting tumor cells and minimizing impact on normal tissue, these studies offer novel avenues for future cancer treatments and instill hope in patients. However, further validation through additional studies is required to advance the safety and efficacy of these treatments in clinical practice and thus benefit a larger population of cancer patients. Simultaneously, these studies furnish valuable data for combining OVs with other drugs to treat tumors, underscoring the potential of this treatment strategy to enhance therapeutic efficacy and overcome drug resistance. Nonetheless, further research and clinical trials are necessary to validate these preliminary findings and determine the optimal course of treatment.

## 5 Conclusion and discussion

OV therapy is an innovative approach for cancer treatment, utilizing viruses to infect tumor cells and induce their death in order to inhibit tumor growth. OVs gene engineering therapy has gained significant attention and research as a potential strategy for treating tumors. This paper provides a comprehensive review and analysis of the engineering modifications, combination therapies, and clinical research involving OVs, aiming to explore its prospects and challenges in tumor therapy.

We have observed that in addition to the aforementioned four strategies, engineering OVs also possess various approaches for enhancing the therapeutic efficacy against tumors. For instance, by utilizing specific functional proteins or enzymes, it is possible to augment the antitumor effect. This finding holds promising implications for the potential utilization of engineered OVs in cancer immunotherapy. However, it is important to note that extensive theoretical research support as well as rigorous animal experiments and clinical trials are still required to further develop and validate this approach.

At the same time, in combination therapy, the combination of OVs with chemotherapy does not consistently yield positive results and may have a detrimental impact on tumor immunoviral therapy. Furthermore, there is limited research on OV combined with chemoradiotherapy; however, existing studies demonstrate its significant potential. This suggests that we can potentially mitigate the side effects of chemoradiotherapy through engineering modifications of OVs and achieve enhanced synergistic effects. Additionally, we are concerned about potential antagonistic mechanisms between OVs and CAR-T therapy based on preclinical studies. Consequently, further investigation into their interaction is warranted in order to optimize this combination therapy regimen. Furthermore, TILs play a pivotal role in this context as well. OV therapy not only directly eliminates tumor cells when combined with TILs but also activates TILs and enhances their immune response against tumors. This enhanced immune response contributes to improvements in the TME by increasing T cell infiltration and activity, ultimately bolstering the immune system’s ability to combat tumors. In the realm of ultrasound-targeted therapy, while microbubble inertial cavitation can significantly enhance the delivery efficiency of drugs or gene carriers, it also presents some inevitable collateral damage, such as microvascular leakage, capillary damage, and erythrocyte extravasation leading to local edema and inflammation. Therefore, before ultrasound-mediated OV delivery can progress to clinical trials, further research is necessary to optimize this technology and minimize its side effects. Despite being in the early stages with limited studies, ultrasound-mediated MB delivery combined with OVs has shown considerable potential, not only for OVs but also for other viral therapies, significantly enhancing therapeutic outcomes and overcoming known barriers.

In summary, OV therapy represents a promising and innovative approach for treating tumors. Through ongoing refinement of engineering strategies, exploration of combination therapies, and clinical studies, we can further enhance the safety, efficacy, and targeting capabilities of OVs to improve treatment outcomes and quality of life for cancer patients. Future clinical applications of OV combination therapies hold significant promise. The potential for synergistic effects, particularly with chemoradiotherapy, offers new avenues for overcoming resistance and achieving more durable responses. However, challenges such as understanding the complex interactions between OVs and immune cells, as well as managing potential antagonistic effects with CAR-T cells, require meticulous research. Prospective studies must focus on optimizing dosing regimens, sequencing of therapies, and patient selection criteria to maximize benefits while minimizing risks. Moreover, the integration of advanced genetic engineering techniques could enhance OV specificity and reduce off-target effects, paving the way for personalized cancer therapies. Despite these advancements, potential obstacles in the clinical environment include regulatory hurdles, high development costs, and the need for large-scale manufacturing capabilities. Addressing these challenges will be critical for the successful translation of OV therapies from bench to bedside. The development of standardized protocols and robust clinical trials will be essential to establish the therapeutic efficacy and safety profile of these innovative treatments. Through continued interdisciplinary collaboration and technological advancements, the future of OV combination therapies appears promising, with the potential to significantly improve cancer treatment.

## Author contributions

ZY: Writing – review & editing, Writing – original draft, Visualization, Investigation, Conceptualization. YPZ: Writing – review & editing, Writing – original draft, Visualization, Investigation, Conceptualization. XW: Writing – review & editing, Writing – original draft, Investigation. XYW: Writing – review & editing, Writing – original draft, Investigation. SR: Writing – review & editing, Writing – original draft, Investigation. XH: Writing – review & editing, Writing – original draft, Investigation. JHS: Writing – review & editing, Visualization. AZ: Writing – review & editing. SG: Writing – review & editing. YC: Writing – review & editing. SD: Writing – review & editing. XuW: Writing – review & editing. ML: Writing – review & editing. FD: Writing – review & editing. YSZ: Writing – review & editing. JS: Writing – review & editing. ZW: Supervision, Conceptualization, Writing – review & editing. ZX: Writing – review & editing, Supervision, Funding acquisition, Conceptualization.
